# An Investigation on Integral Emotions as Parallel Predictors for Risky Financial Behavior

**DOI:** 10.1002/pchj.70089

**Published:** 2026-03-12

**Authors:** Miriam Rustam, Agnes Sianipar, Bagus Takwin

**Affiliations:** ^1^ Faculty of Psychology Universitas Indonesia Depok Indonesia

**Keywords:** anticipated emotion, anticipated outcome, anticipatory emotion, decision‐making, risky financial behavior

## Abstract

This study integrates emotion and decision‐making theories in consumer finance to examine how integral emotions (emotions induced by the decision‐making process) shape risky choices. The purpose of the study is to investigate how integral emotions, specifically anticipatory (felt before deciding) and anticipated (predicted post‐outcome) emotions, work in parallel to influence risky behavior. Unlike prior work that isolated a single emotional pathway, this research offers novelty by modeling both emotional pathways as parallel mechanisms induced by anticipated outcomes, and by quantifying their direct and indirect effects within the same model across two financial contexts. A sample of 640 Indonesians (aged 21–35; 61% female) viewed audiovisual vignettes for “Buy Now Pay Later” (BNPL) and “Online Loan,” then rated perceived future gain/loss (anticipated outcome), the intensity of anticipated and anticipatory emotions, risk perception, intention to use the financial schemes, and completed the risk propensity scale. Path analyses showed that anticipated outcomes robustly elicited both emotion types, and that direct effects of both emotions on risky intention exceeded indirect effects in both contexts. These findings demonstrate that integral emotions influence risky financial intention directly and in parallel, underscore the value of jointly modeling anticipatory and anticipated emotions in risky decision‐making.

## 1 Introduction

Risky financial behaviors are undesirable behaviors that involve the use of money that have negative consequences, such as borrowing money and not paying credit on time (Xiao et al. 2013), which can lead to issues not only related to monetary impact but also affect the subjective and financial well‐being (Sabri et al. 2023). Therefore, it is crucial to understand the variables that drive individuals to engage in risky financial behavior. Emotions are widely acknowledged to significantly shape risky behaviors across various domains, including financial behavior. While emotion reliably shapes risk‐taking, most financial studies investigate the influence of incidental emotions or emotions that are unrelated to the decision at hand on financial decisions (e.g., weather or prior events; Marini 2023). Although proven to influence financial decisions, the fact that incidental emotions are unrelated to decision making makes it difficult to determine whether these emotions are the underlying factors in risky decisions (Ferrer and Ellis 2021).

Instead, we focus on integral emotions—emotions elicited by the decision's options, stakes, and outcomes (Loewenstein and Lerner 2003). Hence, integral emotions are measured when participants are presented with a risky situation and are prompted to consider the available options and their consequences within that specific context (Ferrer and Ellis 2021). This is an important distinction between incidental and integral emotions because the latter are inherent in risky situations. Thus, focusing on integral emotions would be a more straightforward approach to clarifying the underlying mechanism of risky behavior.

Within integral emotions, theory distinguishes between anticipatory emotions, which are experienced at present when decision makers are contemplating options, and anticipated emotions, which are predictions of future feelings post‐decision outcomes (Loewenstein et al. 2001; Schlösser et al. 2013). Thus, anticipatory emotion is a real emotion that is experienced at present due to thinking about the specific risky situation and possible outcomes from the available options, whereas anticipated emotion is the imagination of a future emotion (Baumgartner et al. 2008; Bettiga and Lamberti 2020; Loewenstein et al. 2001).

Separately, anticipatory and anticipated emotions have been shown to predict risky behavior (Lerner et al. 2015), including risky financial behavior (Duxbury et al. 2020; Reimann et al. 2014). The influence of anticipated emotions on risky behavior is based on the consequentialist perspective, which suggests that individuals are motivated by the emotions they expect to experience in the future as a result of their actions (Baumgartner et al. 2008; Schlösser et al. 2013). In this view, consequentialists regard emotions solely as outcomes and do not consider the emotions felt during the decision‐making process (Loewenstein et al. 2001). Loewenstein et al. challenged this perspective using the Risk‐as‐feelings framework. They argue that anticipatory emotions affect the decision‐making process because individuals experience these emotions while considering their options and the consequences of a risky situation (Loewenstein et al. 2001). These differing viewpoints have led to several studies measuring both types of emotion to determine which has the greatest influence on the occurrence of risky behavior.

However, there is mixed evidence regarding which integral emotion matters more. Some studies have indicated stronger anticipatory effects (Schlösser et al. 2013; Young et al. 2019), whereas others have reported stronger anticipated effects (Charpentier et al. 2016). In addition, some studies have suggested outcome‐contingent patterns (Duxbury et al. 2020; Grimani et al. 2024). Recent accounts have proposed that both emotions may be concurrently activated by thinking about outcomes and operating in parallel (Grimani et al. 2024). To the best of our knowledge, few studies explicitly model emotions as parallel processes triggered by anticipated outcomes, and with rare exceptions outside finance (e.g., Barnum and Solomon 2019), even fewer quantify the relative strength of direct versus indirect paths in a single model. We address these gaps by introducing the Parallel Integral Emotion (PIE) framework: anticipated outcomes (expected gains and losses) that elicit anticipatory and anticipated emotions. These emotions simultaneously influence risky financial intentions through either direct or indirect pathways mediated by risk perception.

We aim to address four objectives of this study. First, we investigated whether anticipated outcomes influenced both types of integral emotion, highlighting their parallel nature. Second, we assessed whether anticipatory and anticipated emotions predicted risky financial behavior either directly or through risk perception. Third, we examined the consistency of the parallel mechanism of how integral emotions influence risky behaviors across various contexts. Finally, we explored how individual traits such as risk propensity affect integral emotions.

Our study advances the theory of decision making by positioning integral emotions as parallel predictors of risky decisions, acting directly or indirectly via risk perception. This clarifies the emotional mechanisms underlying risky decision‐making. Moreover, most studies that examine the influence of integral emotions on risky financial decision making employ gambling tasks (e.g., Grimani et al. 2024; Schlösser et al. 2013). Although gambling tasks can be effective in triggering emotions, their ability to reflect real‐life decisions is often challenged. We used audiovisual vignettes of two credit behavior scenarios using “Buy Now Pay Later” (BNPL) and Online Loans to mirror real decisions, integrating emotion and decision‐making theories with consumer finance. In doing so, we advanced both theory and practice by introducing a novel audiovisual vignette method.

## 2 Literature Review and Hypotheses

### 2.1 Anticipatory Versus Anticipated Emotions: Parallel Activation

We anchor our work to the Risk‐as‐feelings framework (Loewenstein et al. 2001), which distinguishes between anticipated and anticipatory emotions and acknowledges their interplay with cognition. The model posits that anticipated emotions tied to anticipated outcomes can evoke anticipatory emotions, shaping the decision‐making process and behavior. Challenging this causal link, Schlösser et al. (2013) argue that anticipated and anticipatory emotions are distinct constructs that may not trigger each other. Subsequent studies (Bettiga and Lamberti 2020; Jäger et al. 2022) have shown that both emotions co‐occur in the same decision context, shaped by thoughts about potential outcomes. Jäger et al. (2022) further emphasized that while both emotions may jointly predict behavior, they do not necessarily cause each other's occurrence.

We followed this view by treating anticipated outcomes as common antecedents of both emotions. As such, anticipatory and anticipated emotions may be generated in parallel when an individual weighs options for decision‐making. Thus, we hypothesized that positive relationships would exist between anticipated outcomes and anticipated emotions (H1a) and anticipatory emotions (H1b).

### 2.2 Direct Versus Indirect Pathways via Risk Perception

The Risk‐as‐feelings framework postulates that anticipatory emotions can directly or indirectly predict risky behavior through risk perception (Hinvest et al. 2021; Loewenstein et al. 2001; Sobkow et al. 2016). Anticipated emotions have also been shown to be capable of directly influencing risky behaviors (e.g., Duxbury et al. 2020) as well as indirectly through risk perception (e.g., Karl et al. 2021). However, few studies have compared these pathways, and none in finance. Barnum and Solomon (2019) found stronger direct than indirect emotional effects on criminal intent; however, neural differences between criminal and noncriminal risk taking (Reyna et al. 2018) limit the generalization of their findings to other risky intentions. Establishing whether direct or indirect effects dominate financial decisions clarifies the emotional mechanism; that is, whether emotions act primarily as immediate drivers of choice or operate through shifts in perceived risk. As such, it guides interventions toward either emotion regulation or risk‐perception recalibration.

Anticipatory emotion functions as a rapid affective signal of threat or safety, and is capable of driving behavior without deliberation (Loewenstein et al. 2001). In contrast, anticipated emotion arises from the cognitive simulation of outcomes and informs risk evaluation, yet can also directly shape choice (Brosch 2021). Previous evidence has shown that one function of integral emotions is to simplify the information received in affective evaluations (Peters et al. 2006). Under outcome uncertainty ingrained in risky decisions, decision makers lean more on affect than analytic appraisals (Rad and Pham 2017). Consequently, integral emotions encode expected outcome values and prediction errors and guide decisions (Asutay and Västfjäll 2022).

Guided by the preceding arguments, we predict stronger direct than indirect (via risk perception) effects of both emotions on risky financial behavior (H2), with this pattern holding across decision contexts (H3). Specifically, anticipatory emotion exerts a significant direct effect across all scenarios (H4a), as does anticipated emotion (H4b).

### 2.3 Individual Dispositions: Risk Propensity

The Risk‐as‐feelings framework also proposes that individual dispositions can modulate anticipatory emotions (Loewenstein et al. 2001), and this is supported by other studies (Bernaola et al. 2021). The same is found for anticipated emotion, although research on the influence of individual dispositions on this emotion is much more limited than on anticipatory emotion (Van Gelder and De Vries 2013). In this study, we considered risk propensity as a potential individual disposition to play such a role in integral emotions. We propose that including risk propensity, which is crucial in explaining risky behavior, will clarify how it influences emotions and help us understand which variable is most responsible for influencing the occurrence of emotions, whether anticipated outcomes or risk propensity.

Risk propensity, which refers to an individual's tendency to approach or avoid risk, drives individuals to pay less attention to objective risk evaluations (Zhang et al. 2020). Although still limited, a few studies have shown that it can trigger anticipatory emotions (Millroth and Frey 2021; Seloni et al. 2023) and influence anticipated emotions (Gabe‐Thomas 2012). These studies suggest that individuals with a higher risk propensity experience more positive emotions in risky situations and are more likely to engage in risky behavior when they disregard risk evaluation (Wang et al. 2016). A high‐risk propensity drives individuals to feel risk as exciting, which drives the decision to choose the riskier option (Gabe‐Thomas 2012; Seloni et al. 2023). Therefore, the final hypothesis was that a significant positive relationship exists between risk propensity and anticipated (H5a) and anticipatory emotions (H5b).

### 2.4 Summary of Hypotheses

**FIGURE 1:**
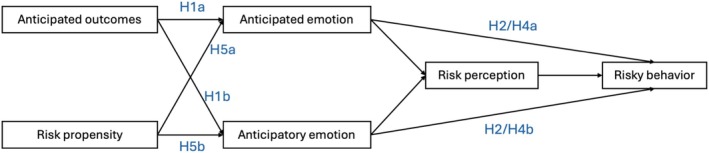
Tested model and hypotheses.

We propose the PIE framework and test the model described. Figure 1 summarizes the model, excluding hypothesis H3. The following hypotheses are tested:

- H1a and H1b: Anticipated outcomes positively predict anticipated and anticipatory emotions.
- H2: The direct effects of both emotions on risky intentions exceed the indirect effects through risk perception.
- H3: These patterns replicate across BNPL and online‐loan contexts.
- H4a and H4b: Risk propensity is positively related to both types of emotion.

## 3 Methodology

### 3.1 Participants

We conducted an online survey using SurveyMonkey. A link was included among the posters distributed on social media, inviting people to participate. Incidental sampling was used, with participants limited to Indonesians residing in Indonesia, aged 21–35 years, with a minimum of high school as their last education, and having a regular income, whether from a part‐time or full‐time job. This age range was selected because individuals aged 18–40 years exhibit increasing levels of risk‐taking, particularly in financial decisions, followed by a subsequent decline (Josef et al. 2016). Within this age range, 19–35 years old is the age group with the highest amount of online credit, which has created well‐being and financial issues in Indonesia. Twenty‐one years of age was chosen as the lowest age limit for participation because it is the lowest age permitted to use online loan platforms in Indonesia, an instrument used in one of the vignettes. Those who completed the survey received an incentive of USD $2. The entire procedure was approved by the Committee on Research Ethics of the Faculty of Psychology, Universitas Indonesia (number: 215/FPsi.Komite Etik/PDP.04.00/2023).

We collected 1038 responses; 148 were excluded because of failed attention checks or incomplete surveys. To meet Amos' path analysis normality criteria (Streiner 2005), we screened for outliers by converting scores to *z*‐scores and removing cases with *z* > 3.29 (Field 2018). The final sample was 640 participants: 61% female, 39% male; ages 21–25 (28%), 26–30 (31%), and 31–35 (41%). Most participants had a high school education (74%). Occupations: private employees, 35%; entrepreneurs, 36%; and others (including homemakers), 29%. Income: 50% below and 50% above Jakarta's regional minimum wage (RMW) (approximately USD 300 at the time).

### 3.2 Vignettes

We used vignettes to represent credit behavior as risky financial behavior. Vignette is effective in triggering emotional reactions (Bran and Vaidis 2019) and is also commonly used by other scholars (e.g., Levidi et al. 2022) when it is difficult to manipulate a real‐world situation for ethical or practical reasons. We developed two vignettes using two different situations: purchasing a gadget using the “Buy Now Pay Later” (referred to as BNPL) scheme on an online shopping platform, and borrowing from an Online Loan platform (referred to as an Online Loan).

In the BNPL vignette, participants imagined wanting to buy a new mobile phone that was better than their current mobile phone. The story explained how much money they had, the regular price of the phone, and a special lower price if they used the “Buy Now Pay Later” plan. It also described the interest rate, monthly payments, and what would happen if they missed a payment, such as extra fees and higher interest. The story also mentioned another phone they could afford now, but it had fewer features.

In the Online Loan vignette, participants imagined that they owned a coffee shop and wanted to expand, but did not have enough money for rent. They had two choices: wait until they saved enough money (but risk losing a good location) or take out an online loan. The story explains the loan's interest rate, monthly payments, and what would happen if they missed a payment, such as extra fees or higher interest.

The vignettes are presented as narrated text in Bahasa Indonesia with visuals (see Videos S1 and S2). We created a voice‐over using listen2it.com and embedded it into visuals and text with Canva. To ensure consistency, the same male voice‐over was chosen for all vignettes. The final files were uploaded to YouTube and embedded into the SurveyMonkey questionnaire.

### 3.3 Measurements

#### 3.3.1 Anticipated Outcomes

Following Sobkow et al. (2016), a single‐item question was used to measure anticipated outcomes by asking, “What are your estimates of the possible returns from doing X?” X refers to risky behavior, that is, buying a gadget using the BNPL scheme and taking online loans. The participants had to answer on a scale ranging from 1 (*very harmful*) to 10 (*very beneficial*).

#### 3.3.2 Anticipated Emotion

Anticipated emotions were measured with the following question: “What do you think you will feel in the future when you get the results of the decision to do X.” X refers to the risky financial behavior depicted in the vignettes. Four typical anticipated emotions in economic and consumer research have been measured: two positive emotions (relief and satisfaction) and two negative emotions (regret and disappointment) (Bagozzi et al. 2016; Wu et al. 2021). An example item is “I will feel regret.” Each emotion was measured using a Likert scale (0 = *not feeling it at all* to 5 = *strongly feeling it*). An index was then calculated by subtracting the average negative emotions from the average positive emotions following the calculation of affect balance (Diener 1984). This calculation is necessary to capture an individual's emotional experience at a given point in time and has been used in various studies (e.g., Veilleux et al. 2020). The Cronbach's alpha for the measures of positive anticipated emotions is 0.82 for BNPL and 0.80 for Online Loan, while for negative anticipated emotions is 0.79 for BNPL and 0.76 for Online Loan.

#### 3.3.3 Anticipatory Emotion

We measured anticipatory emotions with the prompt “How do you feel right now if you decided to do X?” where X is the vignette's risky financial behavior (Grimani et al. 2024; Schlösser et al. 2013). Four typical anticipatory emotions were assessed: two positive (happy, hope) and two negative (anxiety, fear), on a 0–5 Likert scale (0 = *not feeling it at all* to 5 = *strongly feeling it*) (Baumgartner et al. 2008). We formed indices analogous to anticipated emotion indices. Internal consistency was *α* = 0.66 (BNPL) and 0.60 (Online Loan) for positive emotions, and *α* = 0.82 (BNPL) and 0.81 (Online Loan) for negative emotions. Although values around 0.60–0.70 are acceptable, the positive emotion alpha is relatively low, likely due to (i) alpha's sensitivity to scale length (two items) and (ii) the difficulty to evaluate the difference between hope and happiness, which taps related but nonidentical feelings (Cortina 1993; Hair et al. 2010; Taber 2017).

#### 3.3.4 Risk Perception

Severity (“If you decide to do it and you get a bad outcome, how severe do you think it will impact you?”) and likelihood (“How likely are you to get the worst results from doing X?”) were two measures that were adapted to measure the cognitive elements of risk perception (Janssen et al. 2018). Worry (“How worried are you about experiencing the worst results from X”) was measured as an affective aspect of risk perception (Wolff et al. 2019). Each perception was measured using a Likert scale ranging from 1 to 5 (1 = *not severe at all/very unlikely/not worried at all* to 5 = *very severe/very likely/very worried*). Cronbach's alpha for risk perception measures is 0.81 for BNPL and 0.74 for Online Loan.

#### 3.3.5 Risky Financial Behavior

In this study, risky financial behavior was measured by asking participants to what extent they would be likely to engage in the behavior depicted in each vignette. Following Zhang and Shou (2022), a single item was used for measurements. For example, the item used for Online Loan was “How likely will you borrow 20 million rupiahs from an online lending platform to finance your new café?” (1 = *very likely* to 5 = *very unlikely*). This scale was reversed before the analysis.

#### 3.3.6 Risk Propensity

The measurement of the risk propensity was adopted from Dohmen et al. (2017). Two items were used to measure general risk propensity (“Are you generally someone willing to take risks or try to avoid risks?”) and another specifically on financial decisions (“How do you assess your willingness to take risks in matters related to the use of money [e.g., investing, purchasing goods, going into debt, opening a business]?”). Following Dohmen et al. (2017), the scale we used was from 0 to 10, ranging from “*I am not willing at all*” to “*I am very willing*.” Cronbach's alpha for the risk propensity measure was 0.87. Before the analysis, the scale was changed from 1 to 11 to replace the 0.

## 4 Results

We tested the framework (Figure 1) via path analysis using IBM AMOS v23. Pre‐analysis diagnostics were conducted using IBM SPSS v27, including realism checks for vignettes, descriptive statistics, linearity, normality, multicollinearity, and common method bias (CMB).

Vignette realism was rated on a 1–5 Likert scale (1 = not at all; 5 = very close to reality). Both vignettes were judged to be reasonably realistic (BNPL: *M* = 3.46; Online Loan: *M* = 3.16). The relationships between the variables were sufficiently linear. Skewness and kurtosis were < 3 for all variables, indicating approximate normality. Multicollinearity was not a concern (all VIF < 5). CMB, assessed separately for each vignette using Harman's single‐factor test (Kock 2020), showed that the first factor accounted for 37.68% (BNPL) and 40.24% (Online Loan) of the variance, both < 50%, suggesting that CMB is unlikely to bias the results. Table 1 summarizes the pre‐analysis.

**TABLE 1:** Pre‐analysis diagnostics and decision criteria.

Diagnostic	Procedure/metric	Criterion (decision rule)	Result for Buy Now Pay Later	Result for Online Loan	Interpretation
Vignette realism	1–5 Likert (1 = *not at all*; 5 = *very close*)	Mean ≥ 3 indicates acceptable realism	*M* = 3.46	*M* = 3.16	Acceptable realism
Linearity	Bivariate correlation	Sufficient linearity for path modeling	Satisfied	Satisfied	All variables have a linear relationship
Normality (univariate)	Skewness, kurtosis	Less than 2	Less than 2	Less than 2	All have a normal distribution
Multicollinearity	Variance inflation factor (VIF)	VIF < 10 (O'brien 2007)	All VIF < 3	All VIF < 3	No multicollinearity concern
Common method bias (CMB)	Harman's single‐factor test (Kock 2020)	First factor < 50% of total variance	37.68%	40.24%	CMB unlikely to bias results

In the path analysis, two models were generated: one for BNPL and the other for Online Loan. Goodness of fit was assessed using the comparative fit index (CFI), Tucker–Lewis index (TLI), root mean square error approximation (RMSEA), and standardized root mean square residual (SRMR). A perfect model fit is suggested by CFI and TLI values ≥ 0.95, RMSEA and SRMR values ≤ 0.05, whereas CFI and TLI values ≥ 0.90, and RMSEA and SRMR values ≤ 0.08 imply an adequate fit (Hu and Bentler 1999). Four control variables influencing risk perception and risky financial behavior were input directly into the path analysis. These were gender, age, and experience with a particular risky financial behavior, which influence risk perception (Bran and Vaidis 2019; Qian and Li 2020). Age also influences risky financial behavior (Josef et al. 2016), and income influences risk perception in financial contexts (Aydemir and Aren 2017).

We constructed dummy variables for all control variables. Gender was coded as Female = 1 and Male = 0 (reference). Age was categorized into three bands (21–25, 26–30, and 31–35) and dummy‐coded using the 21–25 group as the reference. Two dummy variables were created: one for ages 26–30 and another for ages 31–35 (coded as 1 if in the group, 0 otherwise). Prior experience was coded separately by vignette using dummy variables: Experience_BNPL (1 = previous BNPL use, 0 = otherwise) and Experience_Loan (1 = previous online loan use, 0 = otherwise), with no prior use as the reference category for both. Income was dummy‐coded relative to Jakarta's RMW at the time of data collection (~USD 300/month). Participants were categorized into Income_AboveRMW (1 = monthly income ≥RMW; 0 = otherwise), with those earning below the RMW serving as the reference group.

Model coefficients on these indicator variables are interpreted as differences relative to their respective reference categories in the outcome (risk perception or risky behavior), holding other covariates constant. All vignette models exhibited an acceptable fit based on standard goodness of fit statistics (see Table 2).

**TABLE 2:** Goodness of fit indicators.

Indicators	Buy Now Pay Later	Online Loan
*χ*2	115,002	111,540
df	28	26
CFI	0.97	0.97
TLI	0.95	0.94
RMSEA (90% CI)	0.067 [0.055, 0.080]	0.072 [0.058, 0.086]
SRMR	0.052	0.053

**FIGURE 2:**
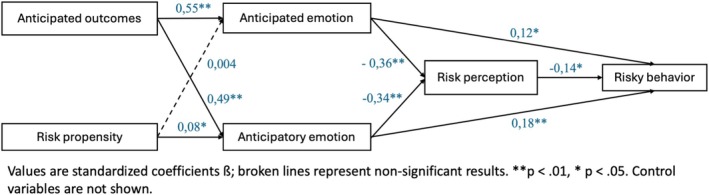
Path analysis model for Buy Now Pay Later.

**FIGURE 3:**
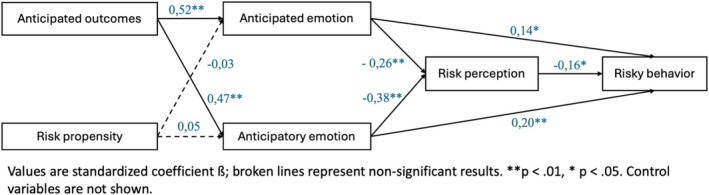
Path analysis model for Online Loan.

Figures 2 and 3 show the models for both vignettes and all coefficients of the relationships. Anticipated outcomes had a positive and significant relationship with both anticipated and anticipatory emotions in all models (in BNPL: *β* = 0.55, *p* < 0.01 with anticipated emotion; *β* = 0.49, *p* < 0.01 with anticipatory emotion; in Online Loan: *β* = 0.52, *p* < 0.01 with anticipated emotion; *β* = 0.49, *p* < 0.01 with anticipatory emotion). Thus, our data support H1a and H1b.

Figures 2 and 3 also show a significant relationship between integral emotions, risk perception, and risky financial behavior. The same applies to the relationship between risk perception and risky behavior. To test whether direct or indirect effects had a stronger role in predicting risky financial behavior, we used the bootstrapping method with *n* = 1000 and a 95% confidence interval. Table 3 shows the direct, indirect, and total effects of anticipated and anticipatory emotions on risky financial behavior for both vignettes. The results showed that the total effects were positive and significant, with the coefficients of the direct effects being larger than those of the indirect effects. These data support H2 and H3. Moreover, the direct effects of both emotions on risky financial behavior were significant. These results support H4a and H4b, respectively.

**TABLE 3:** Indirect (via risk perception), direct effects, and total effects.

	Anticipated emotion		Anticipatory emotion	
	Buy Now Pay Later	Online Loan	Buy Now Pay Later	Online Loan
*β* indirect effects	0.05*	0.04**	0.05**	0.06**
Upper bound CI	0.23	0.08	0.09	0.11
Lower bound CI	0.02	0.01	0.01	0.02
*p*‐value	0.02	0.003	0.005	0.003
*β* direct effects	0.12**	0.14*	0.18**	0.20**
Upper bound CI	0.09	0.27	0.28	0.31
Lower bound CI	0.01	0.03	0.07	0.08
*p*‐value	0.005	0.02	0.003	0.002
*β* total effects	0.17**	0.18**	0.23**	0.26**
Upper bound CI	0.07	0.30	0.33	0.37
Lower bound CI	0.28	0.07	0.12	0.15
*p*‐value	0.001	0.003	0.002	0.002

*Note:* *β* is standardized coefficient.

* *p* < 0.05.

** *p* < 0.01.

The relationship between risk propensity and both emotions was only significant for BNPL, but only between risk propensity and anticipatory emotion (*β* = 0.08, *p* < 0.05). Thus, the data do not support H5a, and H5b is only partially supported. Moreover, the coefficients of the relationship between risk propensity and emotions were smaller than those between the anticipated outcomes for both emotions.

## 5 Discussion

### 5.1 The Main Findings

This study aimed to establish the mechanism of risky financial behavior by testing our PIE framework, in which anticipatory and anticipated emotions were positioned as parallel predictors of risky behavior influenced by anticipated outcomes and risk propensity as individual predispositions. First, we assessed whether the anticipated outcomes were the impetus for integral emotions. We then investigated whether emotions directly predict risky behavior and whether that association consistently exists in different risky situations, that is, “Buy Now Pay Later” (BNPL) and Online Loan. Furthermore, we wish to investigate whether risk propensity as an individual disposition influences both emotions and to determine which variables influence emotions more strongly, that is, anticipated outcomes or risk propensity. Our study is one of the few to consider the possibility that both emotions can occur in parallel, and that the same variables can influence their occurrence. Thus, this study contributes to the theory by formally modeling the co‐activation of both emotion types within a single structural framework and identifying the antecedents responsible for their joint elicitation.

Extant research that also measured both types of integral emotions only emphasized the role of a singular pathway, either anticipatory (e.g., Schlösser et al. 2013; Young et al. 2019) or anticipated (e.g., Charpentier et al. 2016). Thus results are still inconsistent. Although other scholars, such as Duxbury et al. (2020) and Grimani et al. (2024), have proposed that both emotions may play a role in risky financial decisions, none have investigated parallel mechanisms. Our path analyses revealed that anticipated outcomes robustly predicted both anticipatory and anticipated emotions, with comparable magnitudes of influence. These findings extend earlier evidence by empirically specifying anticipated outcomes as common antecedents that produce co‐occurring integral emotions (Bettiga and Lamberti 2020). We then support the conceptual separation from the Risk‐as‐Feelings model's earlier assumption that anticipated emotions are subsumed within expected outcomes (Schlösser et al. 2013). More importantly, since both emotions have a common antecedent, they have the same potential to have a role in influencing the decision process, supporting recent arguments that anticipatory and anticipated emotions should be treated as distinct yet parallel processes (Grimani et al. 2024; Jäger et al. 2022).

A key contribution of this study lies in the consistent pattern that integral emotions exert stronger direct effects on risky intentions than indirect effects through risk perception. Although integral emotions are reliably associated with risk perception, reinforcing that emotions shape cognitive appraisals of risk (Qian and Li 2020; Sobkow et al. 2016), the mediating pathway was weaker than the direct path to intention, a pattern consistent with prior evidence from outside finance (Barnum and Solomon 2019). The Risk‐as‐feelings framework posits that vivid, concrete outcome representations heighten affective responses and their immediacy of choice (Loewenstein et al. 2001). Our use of audiovisual vignettes that provide vivid, temporally dynamic, and sensory‐rich representations likely enhances emotional engagement (compared to static text), amplifying both anticipatory and anticipated emotional responses. This design feature may partly explain why emotional pathways demonstrated robust predictive power even when cognitive mediation via risk perception remained statistically significant.

Our model suggests that anticipatory and anticipated emotions operate concurrently as complementary mechanisms that guide risky decision‐making. Anticipatory emotions act as rapid, affective heuristics—immediate “feeling cues” that simplify complex judgments through intuitive “good–bad” evaluations (Peters et al. 2006; Slovic 2010). These visceral signals (“this feels hopeful” or “this feels risky”) help individuals navigate intricate financial information by bypassing detailed reasoning when time pressure or uncertainty is high. Anticipated emotions, meanwhile, extend this process by transforming imagined future outcomes into cognitive–affective cues that shape current intentions through the simulation of satisfaction, relief, or regret (Carrera et al. 2012; Epstude et al. 2016). Together, they form an integrated emotional system in which anticipatory feelings provide the immediate motivational push, while anticipated emotions offer corrective or regulatory foresight. Both function as heuristic shortcuts rooted in associative experience and affective forecasting (Sobkow et al. 2016), enabling decision makers to act without engaging in laborious risk computation—consistent with dual‐process models of judgment (Rad and Pham 2017; Strack and Deutsch 2004). In online credit behavior, such as using “Buy Now Pay Later” and considering an online loan, the ease to get the goods or money without any collateral, immediate gratification, and urgency increase the possibility for individuals to rely on these affective and cognitive–affective cues to act, lowering self‐control and logical risk considerations (Liu and Zhang 2021; Ma and Yao 2023).

Taken together, our results demonstrate that integral emotions, although empirically separable, contribute concurrently and directly to financial decision making. This reconciles two theoretical positions often treated as incompatible: the consequentialist perspective, which emphasizes anticipated emotion, and the Risk‐as‐a‐feeling perspective, which emphasizes immediate anticipatory affect. Our evidence suggests that these frameworks are complementary rather than competing, reflecting temporally layered processes that jointly shape risky choices. In both vignettes, the relationship coefficients of anticipatory emotion with risky behavior were slightly greater than those of anticipated emotion, indicating that anticipatory emotion had a slightly stronger influence on risky behavior. However, anticipated emotions did not lose their influence on risky behavior. This supports the notion that rather than influencing one another, the two emotions are likely to interact or function in parallel in predicting risky behavior (Grimani et al. 2024; Jäger et al. 2022). We also supported Barnum and Solomon (2019), who posited that integral emotions play a role in triggering intent. What we offer differently from these scholars is that we show this parallel function of integral emotions in non‐gambling, noncriminal risky behavior, thus providing additional support that the parallel role can be extended to other risky decisions.

While the emotional mechanisms are parallel across contexts, with anticipatory emotion exerting a stronger influence, the Online Loan scenario exhibits a marginally tighter anticipatory emotion–intention coupling than BNPL. The total effect of anticipatory emotion was slightly larger for Online Loan (*β*_total = 0.26) than for BNPL (*β*_total = 0.23), with a similar but smaller pattern for anticipated emotion (Online Loan *β*_total = 0.18; BNPL *β*_total = 0.17). This pattern aligns with the Risk‐as‐Feelings account: Higher uncertainty and perceived stakes amplify affective guidance (Loewenstein et al. 2001). We speculate that the uncertainty embedded in the Online Loan vignette stems from the platform's inherent characteristics. The scenario juxtaposes a salient, concrete, and immediate outcome (securing funds to rent the shop) with a more abstract and uncertain one (achieving future business profit). This structure mirrors the real‐world dynamics of online lending platforms, which provide consumers with rapid, low‐friction access to credit that can be used for diverse purposes, including those with inherently uncertain returns. Therefore, by design, such platforms accentuate the tension between the immediacy of financial relief and the ambiguity of long‐term outcomes. Such outcome structure likely privileges anticipatory emotion as a diagnostic signal for choice, while anticipated emotion is attenuated by the difficulty of forecasting distant, ambiguous payoffs. Such findings suggest boundary conditions for affective influence: As outcomes become more abstract and temporally distant, anticipatory emotions dominate decision processes; when outcomes are concrete and easily envisioned, the relative influence of anticipatory emotions diminishes (Jendrusina et al. 2020). This interpretation converges with affective–temporal theories, proposing that concreteness modulates the intensity and temporal alignment of emotional responses (Lerner et al. 2015).

Risk propensity, as an individual predisposition, only had a significant positive relationship with anticipatory emotions in BNPL, but it was not significant for Online Loan. This shows that risk propensity is not a stable, global trait; rather, it should be regarded as context dependent (Frey et al. 2022). Routine, lower‐stakes consumer credit decisions may activate generalized risk tendencies, whereas high‐stakes or novel financial contexts, such as business loans, evoke idiosyncratic emotional reactions dominated by situational cues and perceived consequence severity. The dominance of contextual over dispositional factors in predicting emotional activation highlights that *what people face* in a decision often matters more than *who they are*.

These findings advance emotional decision‐making research by specifying when and how integral emotions operate as direct and parallel predictors of risky intention. Practically, they caution that interventions aimed at changing financial risk behavior should focus less on trait‐level risk tolerance and more on shaping the emotional and cognitive framing of financial outcomes, particularly their concreteness, immediacy, and affective tone.

### 5.2 Additional Analysis: The Direct Effect of Anticipated Outcomes on Risky Behavior

The main focus of this study is to investigate the role of integral emotions in influencing risky financial behavior. To ensure a comprehensive understanding and address both emotional and direct outcome‐based pathways, we conducted an additional analysis to examine whether anticipated outcomes have a direct impact on risky behavior, a relationship we do not consider in our PIE framework. This supplementary analysis allowed us to clarify the extent to which anticipated outcomes independently predict risky behavior beyond the influence of emotional processes.

The results showed that for both vignettes, both direct effects from anticipated outcomes to risky behavior and indirect effects via anticipated and anticipatory emotions are significant. Interestingly, the direct effect of anticipated outcomes on risky behavior was consistently stronger than the indirect effects via both emotions (in “Buy Now Pay Later”: *β*_direct = 0.34, *p* < 0.001 and *β*_indirect = 0.084, 95% CI = [0.031, 0.144], *p* = 0.003; Online Loan: *β*_direct = 0.25, *p* < 0.001 and *β*_indirect = 0.14, 95% CI = [0.078, 0.192], *p* = 0.002). This suggests that, while mediators play a role in explaining the relationship, anticipated outcomes exert a more direct influence on risky behavior.

The result of our additional analysis appears to conflict with our PIE Framework, which is based on the Risk‐as‐Feelings hypothesis, asserting that integral emotions play a strong role in driving risky behavior. We would argue that this is not the case; rather, our results show that the relative influence of cognitive and emotional processes may depend on the context or type of decisions being made. The reflective‐impulsive model (Strack and Deutsch 2004) posits that cognitive processes often dominate in deliberative decision‐making contexts or situations where individuals do not make decisions impulsively. Emotions may play a stronger role in situations where individuals have less knowledge (Berryessa and Caplan 2020) or have to decide in a high‐stress situation (Starcke and Brand 2012). Our data suggest that for deliberative financial decisions, the cognitive path is the primary driver, with the emotional path where integral emotions become mediators between anticipated outcome and behavior intention, serving as a secondary, reinforcing mechanism (Weber and Johnson 2009). Emotion functions as a support or additional factor that makes individuals more inclined to choose a risky behavior. Thus, this additional analysis enriches our understanding of the mechanism of the occurrence of risky behavior that is strongly influenced by the anticipated outcome, either directly or indirectly via integral emotions.

We highlight that the direct effect of anticipated outcomes on risky behavior was stronger in the “Buy Now Pay Later” context (*β* = 0.34) than in the Online Loan (*β* = 0.25). This may reflect the greater salience and concreteness of anticipated outcomes in BNPL transactions, where consumers often make immediate, tangible purchases. The simplicity and positive framing of BNPL products may also encourage individuals to rely more directly on their expectations of outcomes rather than on emotional deliberation. In contrast, online loans may involve more abstract or long‐term considerations, potentially increasing the role of emotional mediation in decision making. This provides a further explanation for the relatively stronger direct effect of anticipatory emotion in Online Loan than in BNPL, that when there is more uncertainty, anticipatory emotion plays a stronger role.

### 5.3 Implications

The strong influence of anticipated outcomes on integral emotion and directly on behavior has some important practical implications. Practitioners in the field of finance, policymakers, and educators must pay close attention to how financial promises are communicated because individuals are easily influenced by expected outcomes. This is important not only in the field of finance but also in other domains of consumer behavior. Careful management of how future outcomes are communicated is needed, such as balancing the communication of both positive and negative consequences, to enable individuals to consider the probability of getting both gains and losses. Emotional appeal may continue to have an impact, and educators should consider fostering emotional resilience. However, without strategies that directly target cognitive evaluations, such interventions are unlikely to be fully effective.

Communication about positive and negative consequences should engage both emotions and rational thinking. This may call for a persuasive approach by convincing the depictions of risks via emotional examples. Using factors that are relevant for individuals, such as the risk to significant others, not only to oneself (Zhang et al. 2017), will need to be considered. This seems to be particularly important for platforms such as online lending, which have two outcomes that can make individuals pay less attention to risks: the immediate outcome (money borrowed) and the end outcome (e.g., business profits). Policies and ways of communicating these outcomes and risks should be given in a concrete way, for example, via an infographic approach, so that uncertainty can be reduced if decision makers are borrowing money for future outcomes. Cognitive risk processing should be encouraged to ensure that individuals are more careful.

### 5.4 Limitations and Opportunities for Future Research

Given the strong influence of anticipated outcomes on both emotions, future research needs to understand how outcomes influence integral emotions and whether different outcomes trigger different emotions. Grimani et al. (2024) stated that gain and loss situations may differently influence anticipated and anticipatory emotions. Further studies can enrich their findings to more clearly establish how gain and loss situations can influence each integral emotion. Moreover, since Grimani et al.'s study was conducted in the context of gambling, we need to investigate whether the influence of gain and loss situations on integral emotions also applies to other risky financial behaviors, as well as in other behavioral domains.

Our study did not systematically look at the attributes of outcomes. Future work should explicitly model and experimentally manipulate outcome attributes, such as concreteness, uncertainty, temporal distance, and stake magnitude, and test their distinct and joint effects on anticipatory versus anticipated emotions, risk perception, and behavior. Multimethod designs (physiology, process tracing, longitudinal updates) and moderator analyses (e.g., numeracy, uncertainty tolerance, literacy, or individual predispositions such as regulatory focus) will clarify when outcomes exert direct “cold” impacts on choice versus when they primarily operate by shaping integral emotions.

There are limitations to our study that should be noted and improved upon in future studies. This cross‐sectional study relied on self‐reported surveys, which may have affected how well the findings translate into real‐life scenarios. Although we attempted to mitigate this issue using vignettes that portrayed realistic situations, both of which were assessed as being close to reality, discrepancies with actual behavior may still occur because of factors such as social desirability, motivations, goals, or incentives for participating in the survey. Future research should employ different methods or approaches to verify the replicability of these findings. Having said this, we are confident that using situations that are close to reality lends more support to the ecological validity of our findings.

Another limitation of our study was the absence of physiological measures of anticipatory emotions. Such measures have been employed by other scholars in the financial domain (e.g., Bettiga and Lamberti 2020; Hinvest et al. 2021). While some studies have used self‐report measurements to gauge anticipatory emotions (Duxbury et al. 2020; Grimani et al. 2024; Schlösser et al. 2013), physiological measurements can provide more objective information regarding anticipatory emotions as feelings that are immediately experienced (Loewenstein and Lerner 2003). Therefore, future studies should combine self‐reporting with physiological measurements, such as skin conductance or measurements in specific brain regions, to obtain the most sensitive anticipatory emotion measurement. Such combined measurements might sharpen the understanding of how anticipatory and anticipated emotions work in the mechanism of risky behavior.

Moreover, the limited age range in our study was intended to investigate risky financial behavior in this age group (Josef et al. 2016). However, this limitation limits the generalizability of our findings. Thus, future studies should expand the age range without forgetting that different age groups have different associations with the outcome values. This opens up another opportunity to explore the impact of age on outcome value perception and how it influences risky behavior.

Finally, we used incidental sampling and social media recruitment, which may have limited the generalizability of our findings and become a potential source of bias. Therefore, future studies should consider using a more robust sampling method and recruiting participants using a more randomized approach to reduce bias and increase generalizability.

## 6 Conclusion

Our study has established the mechanism of risky financial behavior via our PIE framework, which incorporates the views of consequentialists as well as Risk‐as‐feelings in looking at the role of integral emotion. We made two vignettes to represent credit behavior: one to buy a gadget using BNPL, and another to get an online money loan. We created an audiovisual format for both vignettes, which was embedded in the questionnaire, an approach that was rarely used. The most important contribution of our study is that the direct influence of anticipatory and anticipated emotions on risky financial behavior is stronger than the indirect relationship via risk perception. We also showed that anticipatory and anticipated emotions can predict behavior in parallel and are equally strongly influenced by anticipated outcomes, supporting our main proposition in the framework. Such evidence is essential in the theory building of the role of integral emotions, as it clarifies the mechanisms that require further scientific investigation to find solutions to prevent people from engaging in risky behavior.

## Funding

The authors have nothing to report.

## Conflicts of Interest

The authors declare no conflicts of interest.

## Supporting information


**Video S1:** pchj70089‐sup‐0001‐SupplementaryFile1.mp4.

**Video S2:** pchj70089‐sup‐0002‐SupplementaryFile2.mp4.

## Data Availability

The data that support the findings of this study are available from the corresponding author upon reasonable request.
